# Prognostic values of mid-radiotherapy ^18^F-FDG PET/CT in patients with esophageal cancer

**DOI:** 10.1186/s13014-019-1232-1

**Published:** 2019-02-04

**Authors:** Nalee Kim, Hojin Cho, Mijin Yun, Kyung Ran Park, Chang Geol Lee

**Affiliations:** 1 Department of Radiation Oncology, Yonsei Cancer Center, Yonsei University College of Medicine, Seoul, Republic of Korea; 2 Department of Nuclear Medicine, Yonsei University College of Medicine, Seoul, Republic of Korea; 3 Department of Radiation Oncology, Kosin University Gospel Hospital, Busan, Republic of Korea

**Keywords:** Radiotherapy, Esophageal cancer, Metabolic response, Prognosis, ^18^F-FDG, PET/CT

## Abstract

**Background:**

To identify whether early metabolic responses as determined using ^18^F-fluorodeoxyglucose positron emission tomography/computed tomography (FDG PET/CT) during radiotherapy (RT) predict outcomes in patients with esophageal cancer.

**Methods:**

Twenty-one patients with esophageal cancer who received pre-treatment ^18^F-FDG PET/CT (PET1) and inter-fractional ^18^F-FDG PET/CT (PET2) after 11 fractions of RT (median 23.1 Gy, 2.1 Gy per fraction) were retrospectively reviewed. The region of interest for each calculation was delineated using “PET Edge”. We calculated PET parameters including maximum and mean standardized uptake values (SUV_max_ and SUV_mean_, respectively), metabolic tumor volume (MTV), and total lesion glycolysis (TLG). The relative changes (%) were calculated using the logarithmically transformed parameter values for the PET1 and PET2 scans. Multivariate analysis of locoregional recurrence and distant failures were performed using Cox regression analysis. After identifying statistically significant PET parameters for discriminating responders from non-responders, receiver operating characteristics curve analyses were used to assess the potentials of the studied PET parameters.

**Results:**

After a median follow-up of 13 months, the 1-year overall and progression-free survival rates were 79.0% and 34.4%, respectively. Four patients developed locoregional recurrences (LRRs) and 8 had distant metastases (DMs). The 1-year overall LRR-free rate was 76.9% while the DM-free rate was 60.6%. The relative changes in MTV (ΔMTV) were significantly associated with LRR (*p* = 0.03). Conversely, the relative changes in SUV_mean_ (ΔSUV_mean_) were associated with the risk of DM (*p* = 0.02). An ΔMTV threshold of 1.14 yielded a sensitivity of 60%, specificity of 94%, and an accuracy of 86% for predicting an LRR. Additionally, a ΔSUV_mean_ threshold of a 35% decrease yielded a sensitivity of 67%, specificity of 83%, and accuracy of 76% for the prediction DM.

**Trial registration:**

Retrospectively registered.

**Conclusions:**

Changes in tumor metabolism during RT could be used to predict treatment responses, recurrences, and prognoses in patients with esophageal cancer.

**Electronic supplementary material:**

The online version of this article (10.1186/s13014-019-1232-1) contains supplementary material, which is available to authorized users.

## Background

Cancer staging is currently the most important measure for predicting survival as well as for treatment planning. The use of ^18^F-fluoro-2-deoxyglucose positron emission tomography/computed tomography (^18^F-FDG PET/CT) as an imaging modality has been steadily increasing in patients with most types of solid tumors, including esophageal cancer [1]. The use of FDG uptake, which is indicative of tumor metabolism, has also been explored for its prognostic utility. ^18^F-FDG PET/CT-based metabolic parameters such as the standardized uptake value (SUV) and total lesion glycolysis (TLG) have been established and validated as surrogate parameters for predicting survival in patients with esophageal cancer [2, 3].

Additionally, ^18^F-FDG-PET/CT can be used to evaluate treatment response by determining changes in tumor glucose uptake following chemotherapy or radiotherapy (RT). Several studies of patients with esophageal cancer have indicated that tumor metabolic activity after preoperative treatment is strongly associated with histopathologic responses and overall outcomes [4–7]. However, such assessments usually provide information regarding late treatment responses, the main drawback of which is that physicians are unable to provide patients with nonresponding tumors alternative treatment strategies during the early stages of therapy. As such, other studies have demonstrated satisfactory measurements of metabolic parameters after only 2 weeks of induction therapy with acceptable sensitivities and specificities, allowing for the accurate early prediction of response to treatment in patients with esophageal cancer [8, 9]. To that end, ^18^F-FDG PET/CT has been proposed for predicting early responses and potentially allowing a change of individualized treatment; however, this method has not yet been established in routine clinical practice. Determining early responses using ^18^F-FDG PET/CT would allow non-invasive stratification of patients according to their responses and help devise more effective treatment regimens. Most studies of early responses using ^18^F-FDG PET/CT are limited to patients treated with chemotherapy and not RT. Furthermore, due to the radiation exposure during PET scan [10], mid-radiotherapy PET scan has not been widely adopted regardless of its clinical benefit. To implement predictive ^18^F-FDG PET/CT-guided algorithms for patients undergoing RT, the prognostic values of early metabolic responses as well as specific ^18^F-FDG PET/CT-based metabolic parameters ought to be determined in the setting of inter-fractional ^18^F-FDG PET/CT (PET2) after the initiation of RT. Therefore, the aim of this study was to investigate whether inter-fractional metabolic response evaluation using ^18^F-FDG PET/CT could be used as a predictor of RT response in patients with esophageal cancer.

## Materials and methods

### Study population

Patients diagnosed with esophageal cancer who had undergone RT with PET2 between November 2015 and June 2017 were enrolled in this study. Patients were excluded if pre-RT ^18^F-FDG PET/CT (PET1) was either not available or performed at another institution (*n* = 4) or if they did not complete RT (*n* = 2). Ultimately, we retrospectively reviewed the medical records and tumor characteristics of 21 patients, as well as their clinical outcomes. This study was approved by the Health Institutional Review Boards of Yonsei University Hospital (IRB No. 2017-3510-001). The requirement for informed consent was waived owing to the study’s retrospective nature.

### Treatment

Standard treatment at our center consists of 6 weeks of RT with concurrent chemotherapy. Chemotherapy was administered to all patients using a 5-fluorouracil (5-FU) – based regimen except in 3 patients who were medically ineligible (2 underwent RT alone and 1 received uracil/tegafur). Overall, 18 patients were treated with the 5-FU/cisplatin regimen, 2 cycles of which were administered concurrently during RT. Patients had a 4-week break after the completion of RT, and additional maintenance chemotherapy was performed if a medical oncologist determined that the patient’s performance status would allow for it. 5-FU was administered at 500–1250 mg/m^2^ daily as a continuous infusion using a portable electronic pump on days 1–4, while cisplatin was administered at 40–100 mg/m^2^ on day 1 and during RT sessions.

The consistent RT technique used was as follows: The gross tumor volume (GTV) was delineated using simulation CT (3 mm slice thickness) and included the primary tumor and involved regional nodes that were detected via PET and CT fusion using the MIM software (Mim Software Inc., Cleveland, OH, USA). The initial clinical target volume included the GTV plus a margin of at least 4 cm longitudinally and 2 cm radially. The final planning target volume (PTV) was delineated by adding a 0.3 cm margin to both the GTV and clinical target volume based on our institutional image-guidance strategies (PTV1 and PTV2, respectively). A total of 63 Gy in 30 fractions was prescribed to the PTV1 while 54 Gy in 30 fractions was prescribed to the PTV2 using simultaneous integrated boost for patients who underwent definite chemoradiation therapy. For preoperative chemoradiation therapy, a total of 44.1 Gy in 21 fractions and 37.8 Gy in 21 fractions were prescribed to the PTV1 and PTV2, respectively. All patients received intensity-modulated radiation therapy using volumetric modulated arc therapy. Daily pre-treatment imaging using cone beam CT was performed with corresponding position correction before the delivery of each fraction.

### ^18^F-FDG PET/CT method

^18^F-FDG PET/CT scans were performed with a PET-CT scanner (Biograph TruePoint 40; Siemens Healthcare, Erlangen, Germany). Prior to undergoing ^18^F-FDG PET/CT, each patient fasted for a minimum of 6 h before ^18^F-FDG administration, and the plasma glucose level was maintained below 140 mg/dL. ^18^F-FDG was administered intravenously at an approximate dosage of 5.5 MBq/kg of body weight. After a tracer uptake time of median 58 min (range, 45–63 min), patients were subjected to PET/CT imaging; a low-dose, non-contrast CT scan was obtained for attenuation correction. The intrinsic spatial resolution of the system was approximately 5 mm (full-width at half-maximum) in the center of the field of view. Images were acquired from the skull base to the proximal thigh. PET images were reconstructed using a 3D row-action maximum-likelihood iterative reconstruction algorithm. To minimize the negative predictive value caused by non-specific FDG accumulation in radiation mediated inflammatory lesion, we performed ^18^F-FDG PET/CT assessment 2 weeks after the initiation of treatment [11]. The reliability of the early response evaluation 2 weeks after the initiation of treatment has been identified by multiple series; it represents the time course of RT response [8, 12]. The ^18^F-FDG PET/CT examinations were performed within 1 month before the initiation of RT (PET1) and a median 11 (range, 9–12) days after RT (PET2).

### PET metrics

Measurements of metabolic uptake in FDG-avid tumors following pretreatment and inter-fractional scans were compared and evaluated to predict the response to RT. For analysis of PET metrics, all primary tumors were defined as the region of interest and delineated on the PET1 and PET2 scans using PET Edge, a semi-automatic gradient-based delineation method included in MIM software, consistently by a single radiation oncologist (NLK). This method algorithm places the contour boundary at the location where the signal gradient is highest; it has been found to correspond better to pathological specimens than threshold-based methods [13] and has recently been validated in a multi-observer study that showed superiority over the manual and threshold methods in non-small cell lung cancers [14].

The following quantitative features were extracted from the regions of interest of the PET1 and PET2: maximum and mean SUVs (SUV_max_ and SUV_mean_, respectively), metabolic tumor volume (MTV), and TLG. The SUV_max_ was defined as the maximum activity concentration in the tumor/(injected dose/body weight). The SUV_mean_ was defined as the mean concentration of FDG in the tumor/(injected dose/body weight). The MTV was automatically calculated by the software by summing the areas with each 2-dimensional transverse tumor contour and multiplying the values by the corresponding slice thickness. The TLG was calculated by multiplying the SUV_mean_ by the MTV of the tumor [15].

### Statistical analysis

The association between clinical outcomes and PET metrics was evaluated using the chi-square test for categorical parameters and Student’s T-test for continuous parametric parameters. Multiple PET parameters were logarithmically transformed to meet the assumption of linearity on the logit scale. The relative changes (%) were calculated using the logarithmically transformed parameter values for the PET1 and PET2 scans. Locoregional recurrence and distant metastasis were defined as recurrence within and outside the PTV, respectively. The time to events was measured from the date of the first RT administration. The survival curves were estimated using the Kaplan-Meier method and compared using the log-rank test. Multivariate analysis of locoregional recurrence and distant failures were performed using Cox regression analysis. After identifying statistically significant PET parameters for discriminating responders from non-responders, receiver operating characteristics curve analyses were used to assess the potentials of the studied PET parameters; the sensitivity, specificity, accuracy, positive predictive value (PPV), and negative predictive value (NPV) were calculated for an optimal threshold that was determined by providing equal weight to the sensitivity and specificity on the receiver operating characteristics curve. A *p*-value < 0.05 was considered statistically significant. Statistical analyses were performed using SPSS version 23.0.0 (IBM Corp., Armonk, NY) and R version 3.3.0.

## Results

### Cohort characteristics

The patients’ characteristics are shown in Table 1. Most subjects underwent definitive chemoradiation therapy (*n* = 17, 81.0%). The PET2 was obtained approximately 2 weeks (median, 11 days) after the initiation of RT; the median dose at the time of PET2 was 23.1 (range, 18.9–25.2 ) Gy.

**Table 1:** Patient and treatment characteristics

	Number	Percent
Patient characteristics		
Age at treatment (yrs), median (range)	69.2	(46.4–86.8)
Sex		
Female	3	14.3
Male	18	85.7
ECOG PS		
0–1	12	57.1
2	9	42.9
Pathology		
Squamous cell carcinoma	21	100.0
Adenocarcinoma	0	0.0
Site		
Upper thoracic (UI 20–25 cm)	5	23.8
Middle thoracic (UI 25–30 cm)	9	42.9
Lower thoracic (UI 30–40 cm)	7	33.3
Stage		
I	1	4.8
IIB	4	19.0
IIIA	3	14.3
IIIB	7	33.3
IIIC	6	28.6
Treatment characteristics		
Aim		
Definitive	17	81.0
Preop	4	19.0
Concurrent chemotherapy	19	90.5
Chemotherapy regimen (*n* = 19)		
5-Fluorouracil+cisplatin	18	94.7
5-Fluorouracil monotherapy	1	5.3
RT modality		
IMRT	21	100.0
3D-CRT	0	0.0
Median total dose (Gy), median (range)	63	(44.1–69.3)
Median fraction dose (Gy), median (range)	2.1	(1.8–2.1)
Fractions of RT completed before mid-radiotherapy PET (fractions), median (range)	11	(9–12)
Dose of RT completed before mid-radiotherapy PET (Gy), median (range)	23.1	(18.9–25.2)

*Abbreviations: ECOG PS* Eastern Cooperative Oncology Group performance status, *UI* upper incisor, *RT* radiotherapy, *IMRT* intensity-modulated radiotherapy, *3D-CRT* 3 dimensional-conformal radiotherapy

### PET metrics

The median PET1 SUV_max_ and SUV_mean_ were 15.1 (Interquartile range [IQR] 9.9–19.5) and 7.7 (IQR 5.8–11.5), respectively. Furthermore, the median PET1 MTV and TLG values were 10.2 (IQR 7.3–19.0) mL and 96.2 (IQR 39.7–346.3), respectively. The PET metrics were generally lower on PET2 than on PET1; the per-patient relative changes after RT according to ^18^F-FDG PET intensity (SUV_max_, SUV_mean_) and metabolic tumor volume (MTV, TLG) are shown in Additional file 1: Table S1.

### Treatment outcomes

**Fig. 1:**
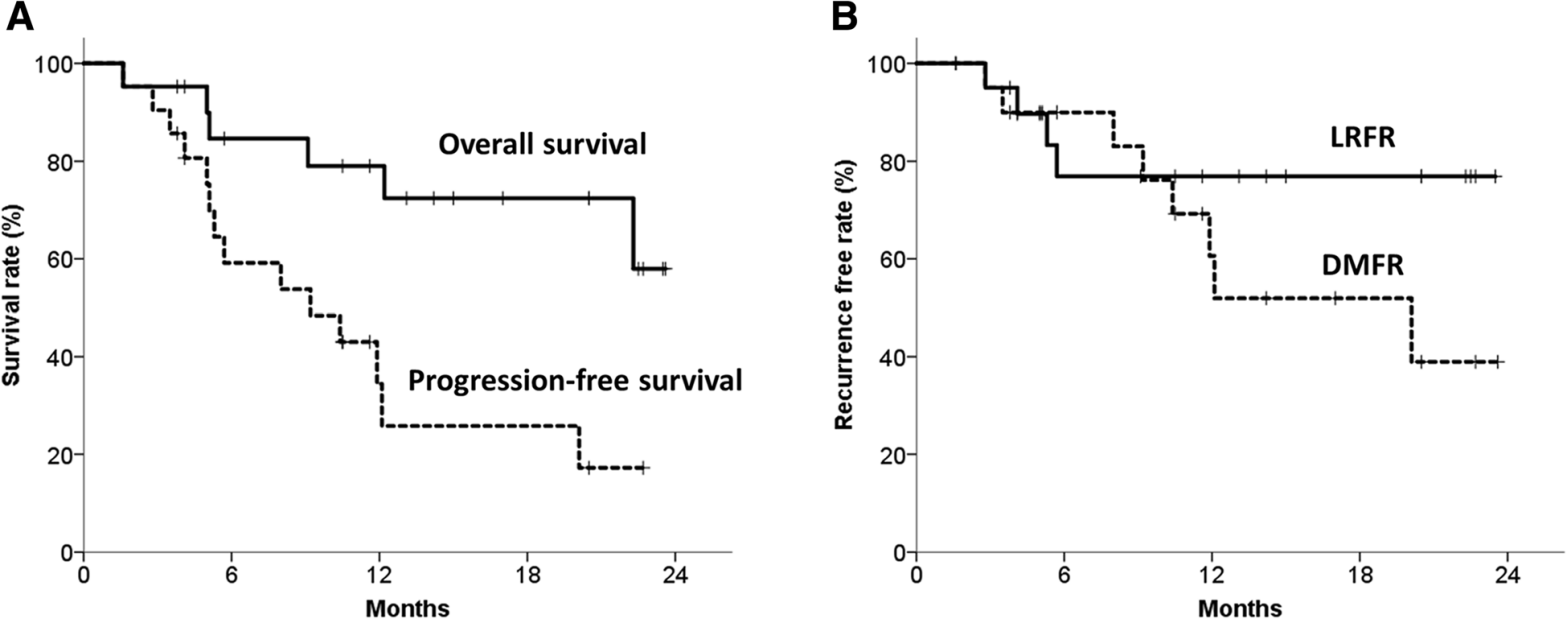
Clinical outcomes for the entire cohort. Progression-free survival, overall survival (**a**), locoregional recurrence-free rate (LRFR), and distant metastasis-free rate (DMFR) (**b**) for the entire cohort

The median follow-up was 13.1 (range, 1.6–23.6) months. The 1-year overall survival (OS) and progression-free survival (PFS) rates were 79.0% and 34.4%, respectively (Fig. 1a). Four patients developed locoregional recurrences and 8 showed distant metastases. The overall 1-year locoregional recurrence-free rate (LRFR) was 76.9%, while the distant metastasis-free rate (DMFR) was 60.6% (Fig. 1b).

### Prognostic PET parameters

The relative changes to the MTV (i.e., ΔMTV) were significantly associated with locoregional recurrence (hazard ratio [HR] 0.98, 95% confidence interval [CI] 0.96–1.00; *p* = 0.03, Table 2). Conversely, the relative changes in the SUV_mean_ (i.e., ΔSUV_mean_) were associated with the risk of distant recurrence (HR 0.90, 95% CI 0.82–0.99; *p* = 0.02, Table 2). However, neither initial GTV, mid-RT GTV, nor relative changes to the GTV was associated with locoregional recurrence or distant metastasis.

**Table 2:** Predictors of locoregional recurrence and distant metastasis identified using a Cox proportional hazards model

	Locoregional recurrence			Distant metastasis		
	Univariate analysis			Univariate analysis		
	HR	95% CI	*p* value	HR	95% CI	*p* value
Patient factor						
ECOG PS (0–1 vs. 2)	4.21	0.44–39.93	0.21	0.47	0.11–1.93	0.29
Stage						
I – IIIA vs. IIIB, IIIC	49.49	0.01–180,664.89	0.35	0.67	0.15–2.94	0.59
PET1						
SUV_max_	0.96	0.81–1.13	0.62	0.95	0.84–1.07	0.37
SUV_mean_	0.89	0.63–1.25	0.49	0.85	0.66–1.09	0.21
MTV^a^	1.00	0.96–1.05	0.84	1.00	0.96–1.03	0.85
TLG^a^	1.00	1.00–1.00	0.93	1.00	1.00–1.00	0.59
GTV^a^	1.00	0.97–1.03	0.84	1.01	0.99–1.03	0.42
PET2						
SUV_max_	1.24	0.95–1.63	0.12	1.08	0.90–1.29	0.42
SUV_mean_	1.92	0.80–4.62	0.15	1.54	0.90–2.65	0.12
MTV^a^	1.02	0.96–1.09	0.48	1.01	0.95–1.06	0.80
TLG^a^	1.01	1.00–1.02	0.29	1.00	0.99–1.01	0.75
GTV^a^	0.99	0.94–1.04	0.64	1.02	0.99–1.05	0.17
Relative difference						
ΔSUV_max_	0.98	0.95–1.00	0.09	0.97	0.94–0.99	0.01
ΔSUV_mean_	0.98	0.95–1.01	0.18	0.95	0.91–0.98	0.00
ΔMTV	0.97	0.95–0.99	0.01	1.00	0.98–1.02	0.76
ΔTLG	0.98	0.96–0.99	0.01	0.98	0.96–1.00	0.04
ΔGTV	1.01	0.97–1.05	0.67	1.00	0.97–1.03	0.82
	Locoregional recurrence			Distant metastasis		
	Multivariate analysis			Multivariate analysis		
	HR	95% CI	*p* value	HR	95% CI	*p* value
Relative difference						
ΔSUV_max_	0.98	0.94–1.02	0.32	1.04	0.98–1.11	0.22
ΔSUV_mean_				0.90	0.82–0.99	0.02
ΔMTV	0.98	0.96–1.00	0.03			
ΔTLG	1.24	0.80–1.56	0.12	1.00	0.98–1.02	0.75

The foreparts of the parentheses were set as the reference groups

*Abbreviations: ECOG PS* Eastern Cooperative Oncology Group performance status, *SUV*_*max*_ maximum standardized uptake value, *SUV*_*mean*_ mean standardized uptake value, *MTV* metabolic tumor volume, *TLG* total lesion glycolysis, *HR* hazard ratio, *CI* confidence interval

^a^Log transformed

### Good responders and non-responders

The optimal ΔMTV and ΔSUV_mean_ cut-off values that discriminated responders from non-responders were calculated as 1.14 (MTV PET2/MTV PET1) and − 35% (i.e., a 35% decrease), respectively.

**Fig. 2:**
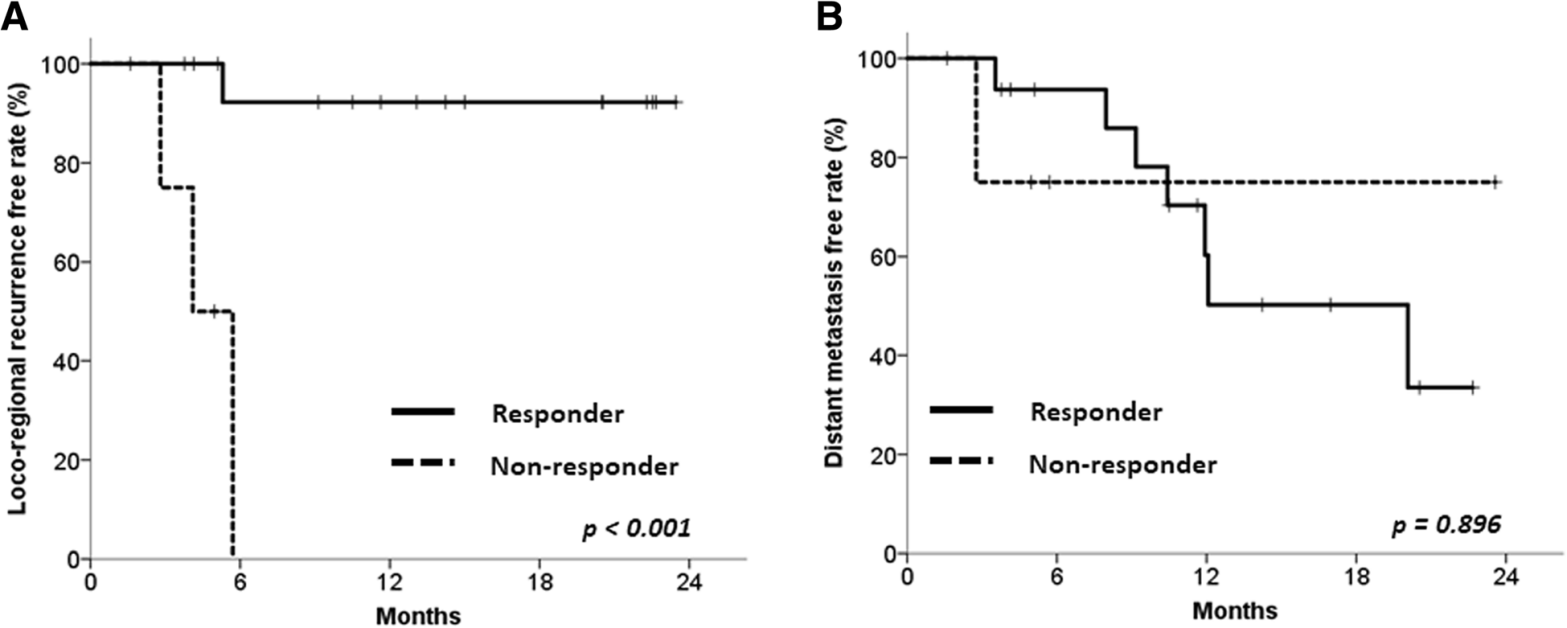
Clinical outcomes according to the MTV change. Locoregional recurrence-free rate (**a**) and distant metastasis-free rate (**b**) of patients according to the MTV reduction ratio (mid-treatment MTV-to-pretreatment MTV). Responders were patients with MTV reduction ratios ≤1.14; while non-responders were patients with MTV reduction ratios > 1.14

The LRFR was more favorable in responders as determined by ΔMTV (*n* = 16, 76.2%) than in non-responders (*n* = 5, 23.8%), with 1-year LRFRs of 92.3 and 0.0%, respectively (*p* < 0.001, Fig. 2a). Distant metastasis was not significantly different between these 2 groups; the 1-year DMFRs were 60.3% vs. 75.0%, respectively (*p* = 0.896, Fig. 2b).

**Fig. 3:**
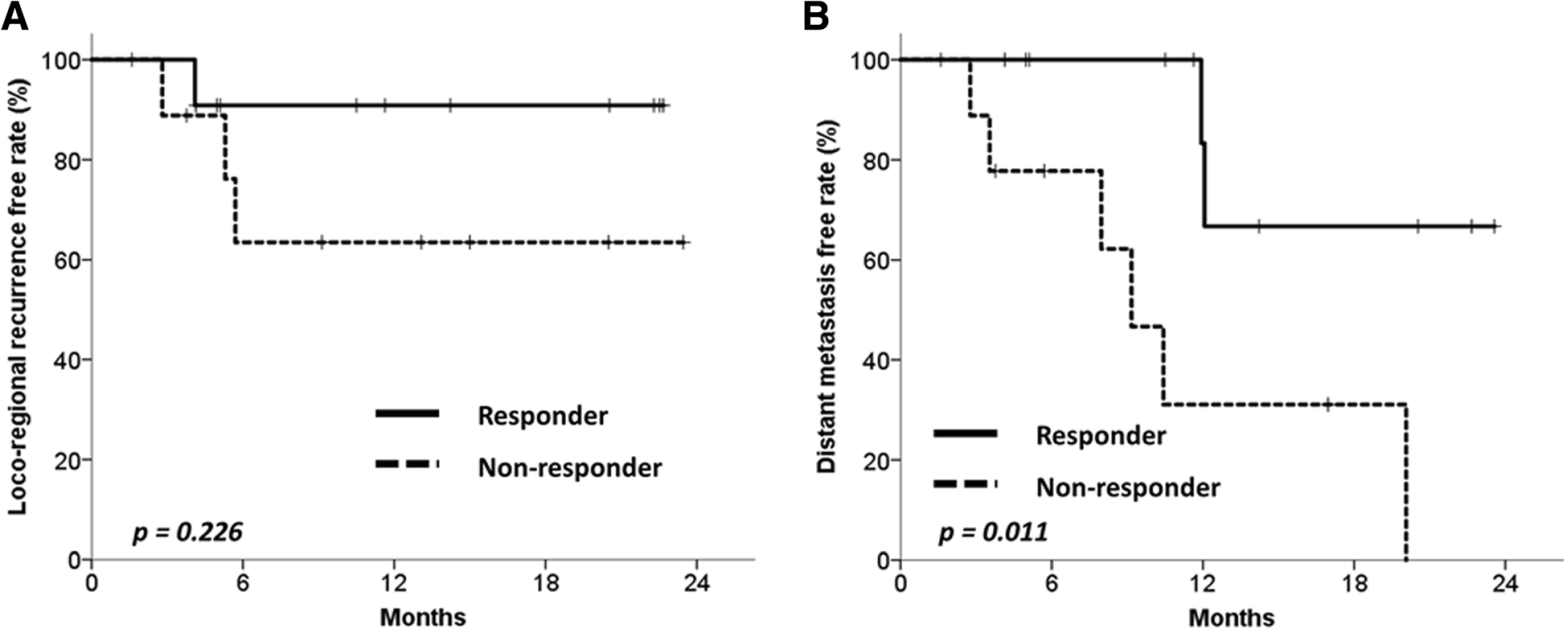
Clinical outcomes acoorindg to the SUV_mean_ change. Locoregional recurrence-free rate (**a**) and distant metastasis-free rate (**b**) of patients according to the mean standardized uptake value (SUV_mean_) reduction rate. Responders were patients with SUV_mean_ reduction rates > 35%, while non-responders were patients with SUV_mean_ reduction rates ≤35%

Furthermore, the DMFR was better in responders as determined by ΔSUV_mean_ (*n* = 12, 57.1%) than in non-responders (*n* = 9, 42.9%); the 1-year DMFRs were 83.3% vs. 31.1%, respectively (*p* = 0.011, Fig. 3b). However, locoregional control was not significantly different between the 2 groups; the 1-year LRFRs were 90.9% and 63.5%, respectively (*p* = 0.226, Fig. 3a).

Based on the ΔMTV criteria, good responders (*n* = 16, 76.2%) showed longer PFS than non-responders (*n* = 5, 23.8%), with 1-year PFS rates of 34.6 and 0%, respectively (*p* < 0.001, Additional file 2: Figure S1). The ΔMTV-based response criteria also exhibited borderline significance for OS, as the 1-year OS rates were 85.7 and 60.0%, respectively (*p* = 0.051, Additional file 2: Figure S1). On the other hand, there were no significant differences in OS and PFS between good responders and non-responders based on the ΔSUV_mean_ criteria (Additional file 3: Figure S2).

### Diagnostic tests

The diagnostic test results are summarized in Table 3. Using the calculated threshold of 1.14, the ΔMTV yielded a sensitivity of 60%, specificity of 94%, accuracy of 86%, PPV of 75%, and NPV of 88% for predicting locoregional recurrence. Furthermore, the ΔSUV_mean_ yielded a sensitivity of 67%, specificity of 83%, accuracy of 76%, PPV of 75%, and NPV of 77% for predicting distant metastasis when the calculated threshold of − 35% was used.

**Table 3:** Diagnostic tests for metabolic response criteria based on MTV and SUV_mean_

	Value (%)	95% CI (%)
MTV - Locoregional recurrence		
Pretest probability	23.8	5.5 – 42.0
Sensitivity	60.0	17.1 – 102.9
Specificity	93.8	81.9 – 105.6
Diagnostic Accuracy	85.7	70.8 – 100.7
Positive Predictive Value	75.0	32.6 – 117.4
Negative Predictive Value	88.2	72.9 – 103.6
SUV_mean_ - Distant metastasis		
Pretest probability	42.9	21.7 – 64.0
Sensitivity	66.7	35.9 – 97.5
Specificity	83.3	62.3 – 104.4
Diagnostic Accuracy	76.2	58.0 – 94.4
Positive Predictive Value	75.0	45.0 – 105.0
Negative Predictive Value	76.9	54.0 – 99.8

*Abbreviations: CI* confidence interval, *MTV* metabolic tumor volume, *SUV*_*mean*_ mean standardized uptake value

## Discussion

We examined the value of using ^18^F-FDG PET metrics before and during chemoradiation in predicting responses to treatment in patients with esophageal cancer. We calculated an optimal cut-off value for ΔMTV (a ratio of 1.14), which was predictive of locoregional recurrence, as well as a cut-off value for the ΔSUV_mean_ (a 35% reduction), which was predictive of distant metastasis. These factors showed satisfactory abilities to predict inadequate responses to RT, indicating that the ΔMTV and ΔSUV_mean_ could be useful for identifying responders.

An accurate prediction of pathologic responses is critical, since patients with poor responses are exposed to unnecessary treatment-related toxicities [16, 17]. Therefore, the predictive value of ^18^F-FDG PET/CT response during treatment has been widely accepted in preoperative chemotherapy for patients with esophageal cancer [9, 12, 18]. Previous studies of ^18^F-FDG PET/CT before and after induction chemotherapy found a significant association between early metabolic response and histopathologic tumor regression [19].

The early response appears to be an indicator of tumor biology and a predictor of the likelihood of treatment failure. As such, early response evaluation can help identify patients who are eligible for treatment intensification or modification, and thus reduce treatment failures in poor responders (the so-called ^18^F-FDG PET/CT guided treatment algorithm). The MUNICON trial prospectively confirmed the feasibility and usefulness of the ^18^F-FDG PET/CT guided treatment algorithm in patients with esophageal cancer. Poor metabolic responders halted chemotherapy and switched to immediate surgery; such early termination of chemotherapy based on metabolic responses did not negatively affect clinical outcome [9, 12].

Tumor stage, nodal stage, and patient characteristics (including morphological information evaluated using esophagoscopy and CT) were not statistically significant prognostic factors on multivariate analysis in our study. On the other hand, the changes in SUV or MTV in the primary tumor, as measured by ^18^F-FDG PET/CT, are dependent on glucose metabolism and reflect the changes in tissue viability in response to chemoradiation. Notably, other series that examined the prognostic value of PET metrics in patients receiving chemotherapy and/or RT for esophageal cancer identified different prognostic metrics, ranging from SUV_max_ [20, 21] to a percentage decrease in TLG [22], the percentage decrease in MTV and TLG [23, 24], and the PET2-based MTV and TLG values themselves [25]. However, there remain statistical concerns such as multiple comparisons and selective reporting of endpoints, because most of these studies were retrospective and examined multiple outcomes. In our series, a small decrease in PET2 MTV was associated with local recurrence, suggesting that this parameter may help select patients that would benefit from further local RT dose escalation. Another promising direction is to consider early switching of systemic chemotherapy in patients with a small decrease in SUV_mean_. Further investigations are required to determine whether additional salvage strategies, such as early switching of concurrent systemic therapy, would be beneficial.

Decrease in MTV represents volumetric changes in the size of high metabolic tumor cells. Thus, it is reasonable to suspect that ΔMTV after local radiotherapy might be a more effective predictor than SUV values in regards to locoregional recurrence [26, 27]. The SUV_mean_ is the SUV value based on MTV. The SUV_mean_ represents the enhanced tapping of ^18^F-FDG into the tumor cells due to biological mechanisms, tumor aggressiveness, and hypoxia [28, 29]. Therefore it provides information of inherent tumor characteristics which suggests a tendency to distant metastasis. In addition, it is well known that the accuracy of CT for assessment of extent in esophageal cancer is limited in the determination of T status [30, 31]. It is the reason why delineated gross tumor volume does not have its prognostication ability.

Our study had several limitations. First, the results should be interpreted with caution because this was a retrospective analysis. Second, ours was a single-center study, which would therefore carry inherent biases. However, our analysis was strengthened by including all possible PET metrics in our diagnostic tests using modern ^18^F-FDG PET/CT techniques and imaging analyses. Other limiting factors include possible inflammatory changes caused by irradiation, which may mimic changes in tumor glucose metabolism associated with treatment effect [32]. Additionally, because of the partial-volume effect, tumor shrinkage upon initiation of treatment may lead to underestimation of the FDG uptake observed on mid-treatment ^18^F-FDG PET/CT scans, and a consequent overestimation of the change of parameters such as the SUV [2]. There is also a possibility of errors introduced by calibration of PET/CT which could the interpretation of SUV make inaccurate [33]. While measuring changes in FDG uptake is a widely adopted parameter that reflects the proportion of viable tumor cells, the development of additional new tracers specific to apoptosis and proliferation may help provide an even more accurate prediction of RT response.

## Conclusion

We found the correlation between decrease in MTV with locoregional recurrence and decrease in SUV_mean_ with distant metastasis, respectively, in esophageal cancer patients treated with chemoradiation therapy. The optimal management of patients with poor responses identified on inter-fractional ^18^F-FDG PET/CT remains to be determined. Furthermore, a prospective study to confirm the efficacy of ^18^F-FDG PET/CT-guided algorithms in patients with esophageal cancer is warranted.

## Additional files

Additional file 1: **Table S1.** Quantitative parameters on the pre-treatment PET1 scan and inter-fractional PET2 scan. (DOCX 17 kb)

Additional file 2: **Figure S1.** Survival outcomes according to the MTV change. Progression-free survival (A) and overall survival (B) of patients according to the metabolic tumor volume (MTV) reduction ratio (mid-treatment MTV-to-pretreatment MTV). Responders were patients with MTV reduction ratios ≤1.14, while non-responders were patients with MTV reduction ratios > 1.14. (TIF 204 kb)

Additional file 3: **Figure S2.** Survival outcomes according to the SUV_mean_ change. Progression-free survival (A) and overall survival (B) of patients according to the mean standardized uptake value (SUV_mean_) reduction rate. Responders were patients with SUV_mean_ reduction rates > 35%, while non-responders were patients with SUV_mean_ reduction rates ≤35%. (TIF 204 kb)

## Abbreviations

^18^F-FDG PET/CT: ^18^F-fluoro-2-deoxyglucose positron emission tomography/computed tomography
DMFR: Distant metastasis-free rate
GTV: Gross tumor volume
IQR: Interquartile range
LRFR: Locoregional recurrence-free rate
MTV: Metabolic tumor volume
NPV: Negative predictive value
OS: Overall survival
PFS: Progression-free survival
PPV: Positive predictive value
PTV: Planning target volume
RT: Radiotherapy
SUV: Standardized uptake value
TLG: Total lesion glycolysis
