# Supplementary material for: Prognostic values of mid-radiotherapy 18F-FDG PET/CT in patients with esophageal cancer
Source: Radiat Oncol. 2019 Feb 4;14:27. doi: 10.1186/s13014-019-1232-1 (PMC6362604; doi:10.1186/s13014-019-1232-1)
Supplement: Supplementary file 1 — Table S1. Quantitative parameters on the pre-treatment PET1 scan and inter-fractional PET2 scan. (DOCX 17 kb) [file 13014_2019_1232_MOESM1_ESM.docx]

**Table S1. Quantitative parameters on the pre-treatment PET1 scan and inter-fractional PET2 scan**

|  | **PET 1** | |  | **PET 2** | |  | **% of decrease** | |
| --- | --- | --- | --- | --- | --- | --- | --- | --- |
|  | **Median** | **IQR** |  | **Median** | **IQR** |  | **Median** | **IQR** |
| GTV | 43.7 | [14.4,58.0] |  | 37.4 | [24.0,58.4] |  | 14.2 | [-3.7,21.3] |
| SUV_max_ | 15.1 | [9.9,19.5] |  | 8.0 | [6.0,10.0] |  | 47.6 | [22.1,59.0] |
| SUV_mean_ | 7.7 | [5.8,11.5] |  | 5.2 | [4.0,5.6] |  | 39.6 | [8.9,54.1] |
| MTV | 10.2 | [7.3,29.0] |  | 11.5 | [5.1,16.4] |  | 27.3 | [4.0,44.0] |
| TLG | 96.2 | [39.7,346.3] |  | 58.0 | [29.7,105.6] |  | 50.4 | [17.2,69.1] |

*Abbreviations:* PET1, pre-treatment ^18^F-FDG PET/CT; PET2, inter-fractional ^18^F-FDG PET/CT; IQR, interquartile range; SUV_max_, maximum standardized uptake value; SUV_mean_, mean standardized uptake value; MTV, metabolic tumor volume; TLG, total lesion glycolysis
