# Supplementary figures and images for: Prognostic values of mid-radiotherapy 18F-FDG PET/CT in patients with esophageal cancer
Source: Radiat Oncol. 2019 Feb 4;14:27. doi: 10.1186/s13014-019-1232-1 (PMC6362604; doi:10.1186/s13014-019-1232-1)

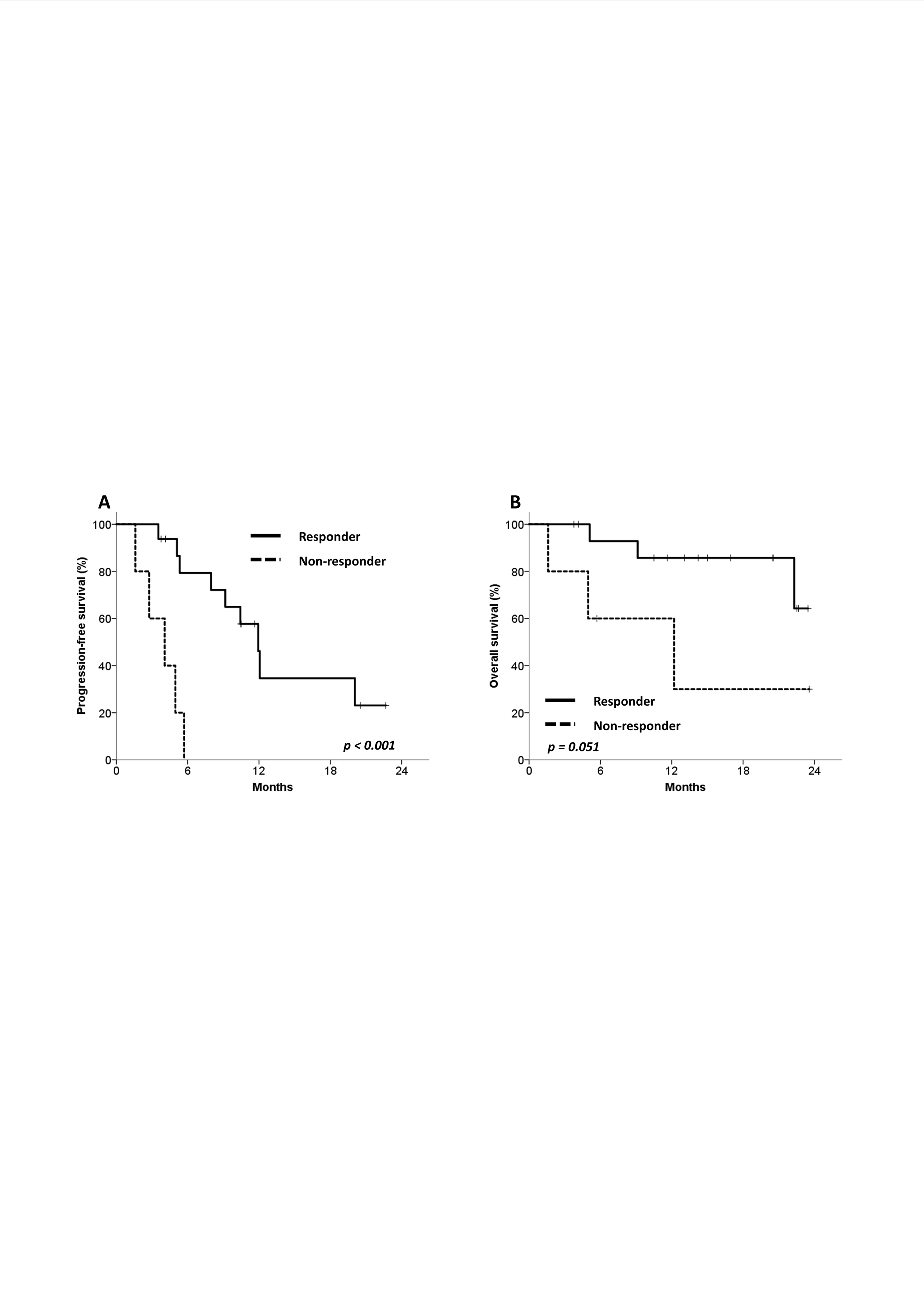

Supplement: Supplementary file 2 — Figure S1. Survival outcomes according to the MTV change. Progression-free survival (A) and overall survival (B) of patients according to the metabolic tumor volume (MTV) reduction ratio (mid-treatment MTV-to-pretreatment MTV). Responders were patients with MTV reduction ratios ≤1.14, while non-responders were patients with MTV reduction ratios > 1.14. (TIF 204 kb) [file 13014_2019_1232_MOESM2_ESM.tif]

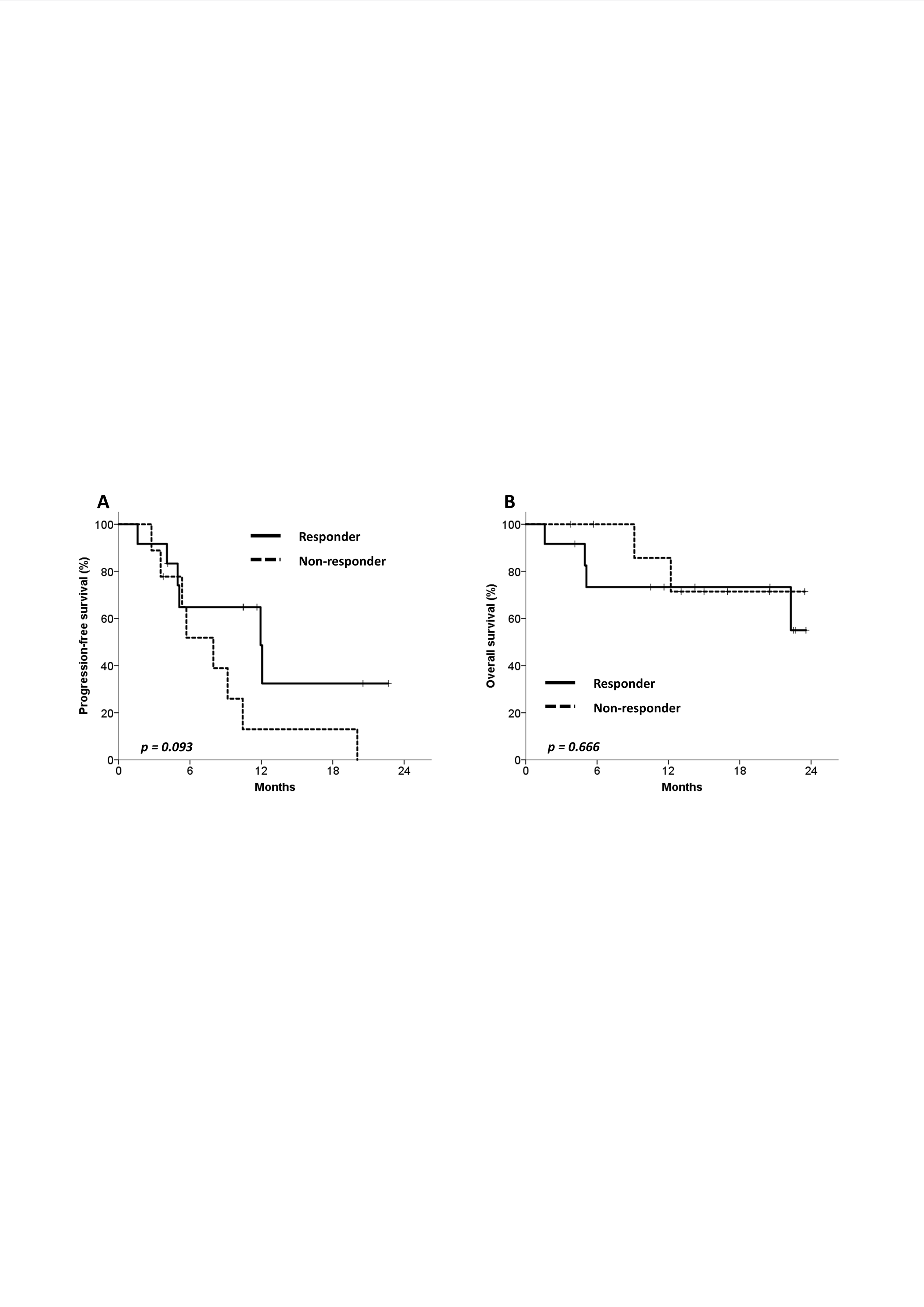

Supplement: Supplementary file 3 — Figure S2. Survival outcomes according to the SUVmean change. Progression-free survival (A) and overall survival (B) of patients according to the mean standardized uptake value (SUVmean) reduction rate. Responders were patients with SUVmean reduction rates > 35%, while non-responders were patients with SUVmean reduction rates ≤35%. (TIF 204 kb) [file 13014_2019_1232_MOESM3_ESM.tif]
